# Introducing *Nursing Reports:* An Open Access Nursing Journal That’s a Little Bit Different

**DOI:** 10.3390/nursrep10010005

**Published:** 2020-09-18

**Authors:** Richard Gray

**Affiliations:** School of Nursing and Midwifery, La Trobe University, Melbourne, VIC 3083, Australia; r.gray@latrobe.edu.au

The purpose of this editorial is to introduce a new publisher and a new editor-in-chief of *Nursing Reports* and set out a mission for the journal. On the 13 July 2020, MDPI took over from PAGEPress as the publisher of *Nursing Reports.* I, Prof. Dr. Richard Gray, became the new Editor-in-Chief on 20 July 2020, taking over from Dr. Colleen E. Marzilli Tyler. I would like to thank Dr. Tyler for her valuable and important contribution to the journal.

By way of introduction, I originally trained as a Mental Health Nurses at King’s College in London, in the early 1990s. I then did my Master’s degree in public health at the London School of Hygiene and Tropical Medicine (2000) and my PhD at the Institute of Psychiatry, King’s College London (2001). I was a Medical Research Council Post-Doctoral Research Fellow 2001 through 2005 and got my first Chair in 2008 at the University of East Anglia, Norwich. I moved to La Trobe University in Melbourne, Australia, in 2017. Over the course of my career, I have published some 220 papers, most of which—to be honest—are rather dull and often contradictory. If, for some peculiar reason, you are interested in reading some of my papers, they are dutifully listed on my Google Scholar page (https://rb.gy/5jennp).

My aim—as Editor-in-Chief—is that, over the next five years, *Nursing Reports* becomes a leading, innovative and progressive open access nursing science journal that publishes rigorous and impactful research and scholarship. In this editorial, I would like to explain how we—the publisher, the editorial board and editorial team—will work with you, as putative authors, to achieve this vision.

## 1. Introducing MDPI

*Nursing Reports* is MDPI’s (Multidisciplinary Digital Publishing Institute) first nursing-specific journal. You may not know much about MDPI as a publisher, and I suspect that you will probably be interested in some background information. Founded in 1996, MDPI is Open Access academic publisher globally and publishes 267 titles, including 73 Science Citation Index Expanded and 1 Social Science Citation Index—Web of Science (Clarivate Analytics)—covered journals [1]. MDPI is the largest Open Access publisher and the 5th largest academic publisher in the world, publishing around 110k articles annually, of which 103k are research manuscripts and reviews [2]. Why should you consider open access publishing? For a start, content is not hidden behind a paywall. Consequently, there is much greater consumer and clinical engagement with your work. Second, open access papers are read and cited more often and finally many major funding bodies—quite rightly—now require that research findings, that are often funded by the public purse, are available to the public that paid for the work. Some authors have legitimate concerns about open access publications; by way of assurance, MDPI is listed as a publisher in the Directory of Open Access Journals (DOAJ) (https://doaj.org/).

## 2. Nursing Reports

One of the defining features of MDPI journals is the speed with which manuscripts are handled. The average time from submission to publication across all MDPI journals is 39 days and the acceptance rate is 40% [2]. These are metrics that matter to authors and no doubt have contributed to MDPI’s rapid grown as a publisher. MDPI is ambitious to see *Nursing Reports* emerge over the next few years as a leading nursing journal that publishes high quality research and scholarship. My main task—and that of my editorial and MDPI colleagues—is to ensure that papers are handled in a timely way, consistent with best practice for journal editors. If you are interested, you can read more about the code of conduct editors are required to follow by using this link to the COPE website: https://rb.gy/lfcr36. I take the COPE code of conduct extremely seriously and if you, as an author, reader or reviewer, feel that my conduct has fallen below these standards, please write and let me know. There are two recommendations from COPE code that I think are particularly important and would like to underscore: reporting negative results and appealing editorial decisions. There is good evidence that studies reporting negative results (this includes empty systematic reviews) are not reported, potentially distorting the evidence base [3]. Journal editors may be less than enthusiastic about publishing negative results because they are less well cited than studies with statistically significant findings [4]. Whilst the COPE code of conduct clearly states that “studies that report negative studies should not be excluded”, this is guidance many editors overlook. This is wrong. I want to encourage you to submit studies with negative (or unexpected) results and assure you that your work will be judged on its methodological merit, not the outcome. Turning next to appealing decisions. We all get things wrong; journal editors are no different and inevitably I will reject a paper that should have been published. The right to appeal an editorial decision, I see as an important part of the scientific process. I therefore welcome authors pointing out when they think an editorial decision is wrong; I will get an editorial colleague to review the decision and see if they agree with me.

As editor-in-chief, there are two further issues that I want to champion in *Nursing Reports*: pre-registration of research and reporting guidelines. Pre-registration of research is intended to prevent publication bias and reduce data fishing (searching a data set to find significant results) [5]. In nursing the rates of pre-registration of research are poor, for example, when it comes to clinical trials, only 1 in 10 are prospectively registered [6]. All studies (observational, experimental and qualitative) can—and arguably should—be pre-registered. It does not take long to do and can be done through the Open Science Framework (https://osf.io/) or clinical trial registries (e.g., https://www.anzctr.org.au/). Another way to pre-register you work is to publish your protocol, there a few nursing journals that publish this type of manuscript. *Nursing Reports* actively encourages authors to submit protocols of their research—systematic reviews, clinical trials, observational and qualitative research. By describing and justifying research methods prior to starting fieldwork or data extraction, we hope to improve the quality of nursing research and scholarship. Of course, I also hope you will submit the results of your research to *Nursing Reports.*

Turning to reporting guidelines: The Equator Network was established some 15 years ago with the aim of promoting transparent and accurate reporting by encouraging researchers to report their research findings against a set of guidelines. Reporting guidelines are structured tools that researchers can use when planning their research and preparing their results for publication. In my editorial experience, few nurse authors make use of reporting guidelines and, as a consequence, papers can be tough to read and process. It will be a requirement of papers submitted to *Nursing Reports* that authors report their research against the appropriate reporting guidelines. I hope you agree this is a positive and progressive position to take that will make research findings more broadly accessible.

## 3. Promoting Dialogue

The publication of a paper in an academic journal is not the end of the scientific process. Post-publication review is essential in confirming observations. In leading medical journals, such as the *Lancet*, 43% of the documents they published in 2019 were letters. In leading nursing journals over the same period, less than 1% were letters. In medicine, the tradition of letter writing is strong. We have all witnessed the power of letter writing in exposing and eventually leading to the retraction of some bad science about the drug hydroxychloroquine during the COVID-19 pandemic. Seemingly, nurses write, and nursing journals publish, few letters. This lack of post publication scrutiny worries me, and I want to actively encourage readers who identify issues with the papers we publish to write and let us know. We need to encourage more dialogue and debate if our research is to be considered credible. For clarity, letters published in *Nursing Reports* do not incur an article processing fee.

## 4. The Editorial Board

*Nursing Reports* is in the process of expanding its editorial board. In my opinion, an engaged and active editorial board is vital to a vibrant and successful journal. I am keen to work with an editorial board that is diverse and inclusive; I would therefore like to extend an invitation to active researchers to apply to join the journal editorial board of *Nursing Reports*. The decision to appoint will be based on enthusiasm and motivation, stage of career, and publication trajectory.

## 5. Holding to Account

How will you know if I and my editorial colleagues are doing a good job? Typically, the performance of a journal is determined by counting citations (impact factor); whilst this metric is clearly important, I want to argue that there are other important quality metrics that should inform potential contributors’ opinion of a journal. These might include: how long it takes from a the submission of a paper to a first decision being made, the proportion of manuscripts that are rejected, how many appeals and complaints an editor receives (and how many were successful), the proportion of papers where study data are available, and the rates of preregistration. In my opinion, grading journals on how well they share information will promote greater transparency and openness in academic publishing. It is my intention to publish an end of year “report card” setting out how *Nursing Reports* is doing against these (and other) quality metrics.

## 6. A Focus on Openness

*Nursing Reports* is a nursing journal that I hope will be a little bit different because of our focus on openness and transparency. I look forward to working with you as authors, reviewers, and readers of the Journal.
